# Frequency of Homocysteinemia in Young Ischemic Stroke Patients and Its Relationship with the Early Outcome of a Stroke

**DOI:** 10.7759/cureus.5625

**Published:** 2019-09-11

**Authors:** Farheen Niazi, Ayesha Aslam, Sadaf Khattak, Satia Waheed

**Affiliations:** 1 Neurology, Pakistan Atomic Energy Commission Hospital, Islamabad, PAK; 2 Neurology, Mayo Hospital, Islamabad, PAK; 3 Internal Medicine, Services Hospital, Lahore, PAK

**Keywords:** homocysteinemia, acute ischemic stroke, risk factors, outcome, stroke in young

## Abstract

Objective

To find out the frequency of hyperhomocysteinemia in young ischaemic stroke patients and its relationship with early morbidity and mortality.

Methods

This prospective study was conducted on young ischemic stroke patients in Pakistan Atomic Energy Commission General Hospital, Islamabad. Ischaemic stroke patients of age < 45 years were selected from both the outpatient and inpatient departments. A fasting venous blood sample was sent for analysis. Data was collected through a structured proforma and were analyzed using SPSS 24.0 (IBM Corp, Armonk, NY, US). The outcome was measured at discharge using the modified Rankin scale.

Results

The mean age of the 71 patients in the study was 35.8 years. Overall, 36 (50.7%) cases had hyper-homocysteinemia. The frequency was significantly higher in males and in the age group 36-45 years (63.4%). Levels of homocysteine did not significantly affect the outcome at discharge.

Conclusion

Hyperhomocysteinaemia, a modifiable risk factor for ischaemic stroke, was seen in about half of young stroke patients. The levels of homocysteine did not correlate with early stroke outcome.

## Introduction

Stroke is the second leading cause of death and the third leading cause of disability worldwide [1]. Strokes occur in about the 15 years younger age group in low and middle-income countries and cause more mortality than in high-income countries [2]. Though the incidence of stroke is falling in the west, it is probably rising in Asia. According to the Global Burden of Disease Study 2010, stroke is the leading cause of disability-adjusted life years (DALYs) in southeast Asia [3].

Epidemiological studies of stroke in the Pakistani population are lacking. Some hospital-based studies have revealed a high proportion of young stroke patients in Pakistan. Khan JA et al. reported in a study that 68/260 (26%) of their patients were 15-45 years of age [4]. Vohra et al. reported that 34% of their patients in stroke case series were under the age of 50 years [5]. Syed et al. reported a frequency of 28% of young stroke under the age of 55 years [6].

The impact of stroke is greatest when it affects young bread earners of the family. The majority of young stroke survivors continue to live with disabilities and remain dependent on families for the costs of rehabilitation and long-term care [1].

Some risk factors for stroke are non-modifiable such as age, gender, and positive family history while other risk factors, such as hypertension, diabetes, and hyperlipidemia, can be managed. Seventy percent of strokes are due to known risk factors [7]. Homocysteinaemia is also one of the modifiable risk factors of stroke [7]. In one study, moderate hyperhomocysteinemia was an independent stroke risk factor seen in 30% of a group of Malaysian ischaemic stroke patients [7].

Studies have also demonstrated that elevated homocysteine levels are associated with higher mortality rates from stroke and coronary heart disease [8]. Thus, this study was conducted to know the frequency of homocysteinemia in our young ischemic stroke patients and whether it has any significant effect on early morbidity and mortality from stroke.

Homocysteine (Hcy) is a four-carbon amino acid formed by the demethylation of methionine, an essential amino acid derived from the diet. Normal total Hcy (tHcy) concentrations range from 5-15 µmol/L in the fasting state. Hyperhomocysteinemia (HHcy) has been classified into moderate (plasma tHcy concentrations of 15-30 µmol/L), intermediate, or high risk (plasma tHcy concentrations of 31-100 µmol/L), and severe (plasma tHcy concentrations 100 µmol/L) [9]. Both acquired and genetic factors can have an impact on plasma tHcy. Male gender, aging, smoking, impaired renal function, and some drugs like corticosteroids and cyclosporine can also cause hyperhomocysteinemia. Genetic causes include classic homocystinuria and C677T homozygote mutation of 5,10-methylenetetrahydrofolate reductase (MTHFR) [9].

Vitamin B12, vitamin B6, and folate are three main cofactors in Hcy metabolism. All of these have a dietary origin. In developing countries, deficiencies in these vitamins are more prevalent and can cause hyperhomocysteinemia and increased risk of stroke [10]. A study conducted on 2,471 Chinese men and women by Hao et al. showed that decreased plasma levels of folate, vitamin B12, and vitamin B6, male gender, and living in urban areas were significantly related to hyperhomocysteinemia [11]. High levels of homocysteine cause oxidative damage to vascular endothelium with the proliferation of vascular smooth muscle and create a prothrombotic environment through its action on platelets, thrombin, and fibrin [12].

Detection of modifiable risk factors like homocysteinemia could result in the better prevention of stroke and can save young patients from getting disabled because of stroke. Most of the data on this is from the western population and very few studies have been done in the Asian or Pakistani population. The genetics, lifestyle, and the dietary habits of the Pakistani population are different from the rest of the world so the objective of this study was to find out the frequency of hyperhomocysteinemia in Pakistani patients with young ischemic stroke and to know whether it has a relationship with early morbidity and mortality.

## Materials and methods

This observational study based on consecutive sampling was conducted in 71 young stroke patients aged less than or equal to 45 years presenting to the neurology outpatient department (OPD) or emergency in Pakistan Atomic Energy Commission General Hospital, Islamabad, between January 2017 to June 2019. Ischemic stroke was defined as a neurological deficit lasting more than 24 hours with either normal computed tomography (CT) of the brain or evidence of infarct on CT brain. The study was conducted on adult patients, with the lower limit of the age of patients being 13 years.

Each patient was evaluated by brain computed tomography (CT) within 24 hours of admission and by duplex ultrasound of extracranial vessels and echocardiography (transthoracic or transesophageal) within the next five post-stroke days. Brain magnetic resonance imaging (MRI) with MR angiography was performed in selected cases.

Informed consent was taken from each patient and the study was approved by the ethical committee of the hospital.

Patients presenting with hemorrhagic stroke, venous sinus thrombosis, or epilepsy were excluded. Patients taking drugs that might affect homocysteine like oral contraceptives or drugs affecting the vitamin B12 or folate metabolisms, such as multivitamins, niacin, methotrexate, tamoxifen, anticonvulsants, bile acid sequestrants, or nitrous oxide anesthesia, were also excluded. 

An overnight eight-hour fasting venous blood sample was sent to the Armed Forces Institute of Pathology, Rawalpindi, or Shifa International Hospital, Islamabad, for analysis. The data were collected through a structured proforma and were analyzed using SPSS 24.0 (IBM Corp, Armonk, NY, US). The early outcome was measured at discharge using the modified Rankin scale. Most patients were discharged within a week of stroke onset and admission. Patients were only given aspirin at presentation. Thrombolysis was not given using tissue plasminogen activator as the facility was not available.

Frequencies and percentages were calculated for categorical variables, including gender. Mean ± standard deviation (SD) was computed for age and homocysteine values. The type of stroke according to the TOAST (Trial of Org 10172 in Acute Stroke Treatment) classification was also documented [13]. The outcome was measured using the modified Rankin scale at discharge. We also documented the association of homocysteine levels across age groups, gender, type of stroke, and outcome using the t-test and analysis of variance (ANOVA).

## Results

The mean age was 35.93±10.01 years, whereas the mean homocysteine levels were 22.76±12.67 mmol/l. Of the total, 47 (66.2%) patients were males and 24 (33.8%) were females. Of the total, 45 (63.4%) were in the age group 36-45 years. There were 35 (49.3%) patients having normal homocysteine levels, 21 (29.6%) showed high-risk homocysteine levels while the remaining 15 (21.1%) had moderate homocysteine levels. The risk factors seen were hypertension in 27 (38%) patients, diabetes 07 (9.9%), other risk factors (dyslipidemia or coronary and valvular artery disease) in 22 (16.9%) patients, and no risk factors were seen in 15 (21.1%) patients. While the TOAST classification showed large artery atherosclerosis numbered 25 (35.2%), small vessel disease 14 (19.7%), cardioembolism 07 (9.9%), stroke of other determined etiology 11 (15.5%), and stroke of undetermined etiology was seen in 14 (19.7%) patients as shown in Table 1.

**Table 1:** Age, Gender, Homocysteine Levels, Risk Factors, and Type of Stroke Among Patients

Variables		Frequency	Percentage
Age (Years) 35.93±10.01*	< 18	05	7
	18-25	03	4.2
	26-35	18	25.4
	36-45	45	63.4
Gender	Male	47	66.2
	Female	24	33.8
Homocysteine Levels (mmol/l) 22.76±12.67*	< 15	35	49.3
	15-30	15	21.1
	> 30	21	29.6
Risk Factors	Hypertension	27	38
	Diabetes	07	9.9
	Other	22	16.9
	None	15	21.1
Type of Stroke (TOAST Classification)	Large Artery Atherosclerosis	25	35.2
	Small Vessel Disease	14	19.7
	Cardio Embolism	07	9.9
	Stroke of Other Determined Etiology	11	15.5
	Stroke of Undetermined Etiology	14	19.7
Total		71	100

We further cross-tabulated the homocysteine levels with the age, gender, type of stroke, and outcomes of the patients as shown in Table 2. It was found that there was a borderline significant difference (p-value = 0.05) in homocysteine levels across the ages with high risk among the 36-45 years followed by 26-35 years. Male patients (38.3%) were at a high risk as compared to females (12.5%) followed by a moderate risk among males (23.4%) as compared to females having a 16.7% risk. This difference was statistically significant (p-value = 0.026). It was found that large artery atherosclerosis (40%) had a high risk of homocysteine levels as compared to other types according to the TOAST classification, i.e., small vessel disease (35.7%), stroke of undetermined etiology (28.6%), cardioembolism (14.3%), and stroke of other determined etiology (9.1%). This difference was statistically insignificant (p-value = 0.063). Regarding the outcomes of the patients, higher homocysteine levels were seen in a patient who died, followed by moderate disability (42.9%), then moderate to full recovery (31.6%), followed by full recovery (27.8%), and, finally, slight disability (25%). This difference was statistically insignificant (p-value=0.437). Whereas, among hypertensive and diabetic patients, the hyperhomocysteinemia levels were 44.4% and 14.3%, respectively. This difference was statistically insignificant (p-value=0.171) as shown in Table 2.

**Table 2:** Association Between Age, Gender, Types of Strokes, and Outcomes with Homocysteine Levels of Young Stroke Patients TOAST: Trial of Org 10172 in Acute Stroke Treatment

Variables		Homocysteine Levels (mmol/l)			Total	p-Value
		< 15 (%)	15-30 (%)	> 30 (%)		
Age (Years)	< 18	04 (80)	01 (20)	00	05	0.05*
	18-25	01 (33.3)	02 (66.7)	00	03	
	26-35	12 (66.6)	03 (16.7)	03 (16.7)	18	
	36-45	18 (40)	09 (20)	18 (40)	45	
Gender	Male	18 (38.3)	11 (23.4)	18 (38.3)	47	0.026*
	Female	17 (70.8)	04 (16.7)	03 (12.5)	24	
TOAST Classification	Large Artery Atherosclerosis	09 (36)	06 (24)	10 (40)	25	0.063
	Small Vessel Disease	05 (35.7)	04 (28.6)	05 (35.7)	14	
	Cardio Embolism	05 (71.4)	01 (14.3)	01 (14.3)	16	
	Stroke of Other determined Etiology	10 (90.9)	00 (00)	01 (9.1)	11	
	Stroke of Undetermined Etiology	06 (42.9)	04 (28.6)	04 (28.6)	14	
Patient Outcomes	Full Recovery	07 (38.9)	06 (33.3)	05 (27.8)	18	0.437
	Near Full Recovery	09 (47.4)	04 (21.1)	06 (31.6)	19	
	Slight Disability	15 (62.5)	03 (12.5)	06 (25)	24	
	Moderate Disability	02 (28.6)	02 (28.6)	03 (42.9)	07	
	Moderate to Severe Disability	02 (100)	00	00	02	
	Death	00	00	01 (100)	01	
Total		35 (49.3)	15 (21.1)	21 (29.6)	71	
Risk Factors	Hypertension	09 (33.3)	06 (22.2)	12 (44.4)	27	0.171
	Diabetes	05 (71.4)	01 (14.3)	01 (14.3)	07	
Total		14 (41.2)	07 (20.6)	13 (38.2)	34	

Homocysteine levels among males (25.45±12.61) and females (17.5±11.28) showed a statistically significant difference (p=0.011). The < 18 years age group showed 15.4±6.07 levels, the 18-25 years age group showed 22.57±13.65, the 26-35 years age group showed 19.6±13.24, and patients of age group 36-45 showed 24.86±12.69. These differences between the homocysteine levels of various age groups showed no statistical significance. There was also no statistical significance seen between homocysteine levels and the TOAST classification with large artery atherosclerosis (26.4±13.9 mmol/l), small vessel disease (23.38±10.73 mmol/l), cardioembolism (15.57±9.19 mmol/l), stroke of other determined etiology (15.26±9.96 mmol/l), and stroke of undetermined etiology (25.14±13.1mmol/l). Whereas risk factors regarding hypertension and diabetes showed mean homocysteine levels of 27.02±13.79 and 18.5±12.09. respectively. This difference was also statistically insignificant (t-value=1.487; p-value=0.147) as shown in Table 3.

**Table 3:** Comparison of Homocysteine Levels Between Gender, Age Groups (Years), and Type of Stroke *significant value (p-value<0.05),**t-test value(comparing two means); ***Analysis of variance (ANOVA) value (comparing more than two means)

Variables		N	Mean±SD (mmol/l)	t-test/ANOVA Value	p-value
Gender	Male	47	25.45±12.61	2.60**	0.011*
	Female	24	17.5±11.28		
Age (Years)	< 18	05	15.4±6.07	1.369***	0.260
	18-25	03	22.57±13.65		
	26-35	18	19.6±13.24		
	36-45	45	24.86±12.69		
Type of Stroke	Large Artery Atherosclerosis	25	26.4±13.9	2.340***	0.064
	Small Vessel Disease	14	23.38±10.73		
	Cardioembolism	07	15.57±9.19		
	Stroke of Other determined Etiology	11	15.26±9.96		
	Stroke of Undetermined Etiology	14	25.14±13.1		
Risk Factors	Hypertension	27	27.02±13.79	1.487**	0.147
	Diabetes	07	18.5±12.09		

## Discussion

The study showed a strong association between hyperhomocysteinemia and ischaemic stroke. Half of our ischaemic stroke patients had hyperhomocysteinemia. This frequency is similar to the findings presented in some other studies. One study showed that hyperhomocysteinemia was found in 48% of ischaemic stroke patients [14].

In another study, hyperhomocysteinemia was found in 50% of stroke patients, and stroke patients with hyperhomocysteinemia were found to have multiple infarctions and cerebral microangiopathy as compared to patients with normal serum homocysteine level [15].

In our study, males had higher homocysteine levels than females. Another study also reported that males were found to have higher homocysteine levels than females [11]. We also found that males in the age group of 36-45 years were especially found to be high homocysteine levels. Forty-five out of our 71 (63%) patients were in the age group 36-45 years, 27 out of 45 (60%) had high homocysteine levels in our study. In an Indian study, the difference in homocysteine levels between males and females were statistically insignificant [16].

Controversy still exists over which subtype of stroke is allied to hyperhomocysteinemia [9]. Our study showed that patients with large artery atherosclerosis (40%) had high homocysteine levels as compared to other subtypes. One Chinese study also demonstrated that the association between tHcy levels and all-cause mortality was only significant in the large-vessel atherosclerosis stroke subtype and is not significant in the small-vessel occlusion subtype [17].

Eikelboom et al. reported that tHcy was significantly greater in large-artery and small-vessel strokes as compared to cardioembolic strokes and controls [9]. Tan et al. also showed that increased tHcy was associated with a higher risk of large artery stroke [9].

Major modifiable risk factors were similar to stroke patients elsewhere in the world. The most common risk factors in our stroke patients were diabetes mellitus and hypertension. Syed et al. reported that approximately 77% of their cohort had diabetes mellitus, hypertension, or both [6]. Hypertension was the commonest risk factor in our patients (38%) of the patients. Some studies have reported a relationship between hypertension and homocysteine levels [16]. In our study, the association was not statistically significant. Some other studies have also failed to establish any relation [18]. This further re-emphasizes the need for more research studies to observe the association between homocysteine levels and the traditional risk factors of stroke such as diabetes and hypertension.

It is proposed that hyperhomocysteinemia induces an elastolytic process in the arterial wall. The loss of elastin may lead to the stiffening of the arterial wall, resulting in hypertension. This might be one of the factors by which hyperhomocysteinemia contributes as a risk factor for stroke, although other factors may also exist [19]. In other studies, conflicting results have been found regarding the association between homocysteine levels and diabetes mellitus [20].

It is now widely recognized that B vitamin therapy reduces tHcy levels, which can thus lead to a reduced risk of stroke. Therapy to lower tHcy levels with B vitamins has been found to reduce the risk of stroke, as well as the risk of myocardial infarction in several recent trials and meta-analyses. Studies have shown that vitamin B12 supplementation of even 0.4 mg can lower homocysteine levels by 7%. Moreover, folic acid supplementation (0.5 to 5mg) can reduce homocysteine levels by one fourth [21-22].

Stroke-related mortality in the acute stage has been reported in the range of 11%-30%. Studies have also demonstrated that elevated tHcy levels are associated with higher mortality rates from stroke and coronary heart disease. In one Chinese study, they found that there is a strong, independent association between elevated tHcy levels and the risk of mortality from ischemic stroke [17]. In this study, they also found that elevated tHcy levels during the acute phase of an ischemic stroke significantly predict mortality in Chinese patients [17].

In our study, the levels of homocysteine did not correlate with the outcome. Levels of homocysteinemia did not significantly correlate with early morbidity and mortality. Another study on elderly patients showed that moderate hyperhomocysteinemia (>= 30 μmol/L) was associated with poor functional status at discharge from the stroke unit but not with early death [23].

The limitation of our study was that the sample size in our study was relatively small. Hence, further studies should be done in the young Asian population so that these results can be verified on a larger scale.

## Conclusions

Hyperhomocysteinemia is a potentially modifiable risk factor associated with a significant percentage of ischaemic stroke in the young Pakistani population. Hyperhomocysteinemia did not significantly alter the outcome after an acute ischemic stroke in our young stroke patients.
